# Genistein exerts anti-leukemic effects on genetically different acute myeloid leukemia cell lines by inhibiting protein synthesis and cell proliferation while inducing apoptosis – molecular insights from an iTRAQ™ quantitative proteomics study

**DOI:** 10.18632/oncoscience.120

**Published:** 2015-02-06

**Authors:** Karthik Narasimhan, Yew Mun Lee, Teck Kwang Lim, Sarah Alexandra Port, Jin-Hua Han, Chien-Shing Chen, Qingsong Lin

**Affiliations:** ^1^ Department of Biological Sciences, National University of Singapore, Singapore; ^2^ Department of Medicine, Yong Loo Lin School of Medicine, National University of Singapore, Singapore; ^3^ Division of Hematology and Oncology, School of Medicine, Loma Linda University, Loma Linda, California, United States of America

**Keywords:** Acute myeloid leukemia, genistein, mTOR, quantitative proteomics

## Abstract

Acute myeloid leukemia (AML) is a form of cancer that affects the hematopoietic precursor cells with lethal effects. We investigated the prospect of using genistein as an effective alternate therapy for AML. A two-cell line model, one possessing the FLT3 gene with the ITD mutation (MV4−11) and the other with the wildtype FLT3 gene (HL−60) has been employed. Our 8−plexed iTRAQ™−based quantitative proteomics analysis together with various functional studies demonstrated that genistein exerts anti-leukemic effects on both the AML cell lines. Genistein treatment on the AML cells showed that the drug arrested the mTOR pathway leading to down−regulation of protein synthesis. Additionally, genistein treatment is found to induce cell death via apoptosis. Contrasting regulatory effects of genistein on the cell cycle of the two cell lines were also identified, with the induction of G2/M phase arrest in HL-60 cells but not in MV4−11 cells. Hence, our study highlights the potent anti-leukemic effect of genistein on AML cells irrespective of their genetic status. This suggests the potential use of genistein as an effective general drug therapy for AML patients.

## INTRODUCTION

Acute myeloid leukemia (AML) is the cancer in leukocytes of myeloid lineage, distinguished by uncontrolled proliferation of hematopoietic precursor cells with decreased rate of self-destruction and impaired differentiation. It comprises of a heterogeneous spectrum of malignancies classified into various genetic abnormalities and clinical characteristics, leading to variable outcomes by current treatments: 20-75% success rates in patients younger than 60 years and < 10% in elderly patients [1–3].

Current treatment strategies for AML involve chemotherapy, which combines therapeutic agents such as cytarabine (ara-C) and anthracycline (daunorubicin or idarubicin), resulting in 50-75% of adults with AML to achieve complete remission [4]. However, profound myelosuppression, heavy side effects, frequent cancer relapse, poor therapeutic response, and development of resistance to the administered drugs are common deterrents to current treatment strategies. Only 20-30% of patients enjoy long-term disease-free survival [4] and five-year survival for patients under 60 years old is only 40% [5]. Hence, there is a continual need to identify a drug with better efficacy and reduced side effects for treatment of AML.

Prominent among the mutations that characterize AML is the Fms-Like Tyrosine kinase 3-Internal Tandem Duplication (FLT3-ITD) mutation, which accounts for ~23% of AML cases, and is linked to a particularly poor prognosis [6–8]. This mutation renders the FLT3 kinase constitutively active [9]. The presence of FLT3 mutation represents a negative prognostic factor for AML patients [10]. This has driven researchers to develop drugs to specifically target the FLT3-ITD mutation.

FLT3 inhibitors are starting to show a degree of efficacy in clinical trials, with small molecules tyrosine kinase inhibitors leading the way to care for a large number of AML patients in the future [11]. However, most of these FLT3 specific drugs are ineffective against cells without the FLT3-ITD mutation [12, 13]. Therefore, a drug which can potentially exert potent anti-leukemic effects against majority of the AML subgroups would greatly improve the efficacy of AML therapy for many patients. This would reduce the occurrence of ineffective treatments and avoid unnecessary cost.

Genistein is a soybean derivative from the isoflavone family of phytochemicals. It occurs naturally in large quantities in soybean, and one gram of soy protein contains as much as 250mg of genistein. Genistein is a potent protein tyrosine kinase (PTK) inhibitor [14, 15], with little or no effect on serine and threonine kinases. A pioneering epidemiological study on Singapore Chinese women by Lee *et al.* [16] showed that soy consumption directly correlated with reduced risk of breast cancer. Genistein has also been known to exert anti-proliferative effects on several other cancer types [17, 18]. Genistein's anti-leukemic activity was identified in the beginning of the previous decade and has been suggested as an alternative therapeutic option for hematological malignancies [19]. Hence, genistein could be a promising treatment for AML patients.

Due to the multi-targeted PTK inhibiting properties of genistein, a profound effect on the proteome of cancer cells would be expected. Genistein has been found to inhibit proliferation via cell cycle arrest and induce apoptosis in acute lymphoblastic leukemia [20]. However, the detailed anti-oncogenic mechanism of genistein remains unclear. There have been previous attempts to elucidate the regulatory effects of genistein on leukemic cells by mapping its proteomic alterations using a 2D-gel-based approach [20], but the study suffers from the inherent limitations of 2D-gel-based approach, and the results remain inconclusive.

Quantitative proteomics approaches are valuable tools to obtain a snapshot of the protein abundance changes caused by drug treatments and can be used to characterize their mechanism of action. Such a method has been exploited to identify protein alterations from genistein treatment on gastric cancer cells [21, 22]. In our study, using high-throughput 8-plex iTRAQ™ based proteomics approach, we have comprehensively characterized the anti-leukemic effects of genistein in AML cells using two different cell lines: one containing an inherent FLT3-ITD mutation in the FLT3 gene (MV4-11) and the other with the wild-type FLT3 gene (HL-60). We found that genistein exerts anti-leukemic effects on both cell lines, thus highlighting the potential of genistein as a generic AML therapy.

Moreover, our study indicated that several biological functions including protein synthesis, cell cycle and cell death were modulated by genistein. Genistein was also found to regulate critical signaling pathways such as mTOR. Our functional studies demonstrated that genistein inhibits the mTOR pathway and blocks protein synthesis in the two AML cell lines. In addition, our study found that the cell cycle arrest caused by genistein differed in the two cell lines tested. Occurrence of cell death via apoptosis in both AML cell lines were identified as well. In summary, our study provides a comprehensive empirical model for the anti-leukemic effects of genistein on AML harboring different genetic mutations, thus demonstrating the potential of genistein as a general treatment for majority of AML patients.

## RESULTS

### Genistein exerts cytotoxicity on AML cell lines

In our study, we showed that genistein exhibited cytotoxicity on both MV4-11 and HL-60 cells *in vitro*. MV4-11 cell line was found to carry an ITD mutation in the FLT3 gene and is widely used as a model cell line for FLT3-ITD signaling [25], while the HL-60 cells possess the wild-type FLT3 gene [26]. As illustrated in Figure 2A, the cytotoxicity of genistein increases with increasing dosage. The IC_50_ dosage of genistein after 48hr treatment for MV4-11 cells was found to be 20μM, while that of HL-60 was a close 30μM. In the time dependent study using the respective IC_50_ for each AML cell lines, the cytotoxicity of genistein starts to increase drastically for MV4-11 cells after the 48hr mark as compare to the gradual increase of cytotoxicity on HL-60. This highlights the enhanced cytotoxicity of genistein on MV4-11 over HL-60.

### Genistein is a FLT3 inhibitor

The fact that genistein is a known broad-spectrum PTK inhibitor led us to examine its effect on FLT3, a tyrosine kinase. Western blotting studies using an antibody specific to the phosphorylated tyrosine residue 591 of the FLT3 gene showed that genistein does in fact have a potent inhibitory effect on the phosphorylation of FLT3 in MV4-11 cells (Figure 3). In the case of HL-60, phosphorylated FLT3 was not detected, consistent with its wild-type status. Thus, it is validated that genistein is a FLT3 inhibitor, as previously reported using other FLT3-ITD cell lines [27]. Both cell lines showed positive response to the drug, thus understanding the mechanism of action of genistein in both cases could provide new information of how genistein exerts its anti-leukemic effects on two cell lines with different genetic backgrounds. In order to achieve this, the proteomes of the cells treated with the drug were studied.

### The 8-plex iTRAQ-based profiling of the proteome level changes induced by genistein

**Figure 1:**
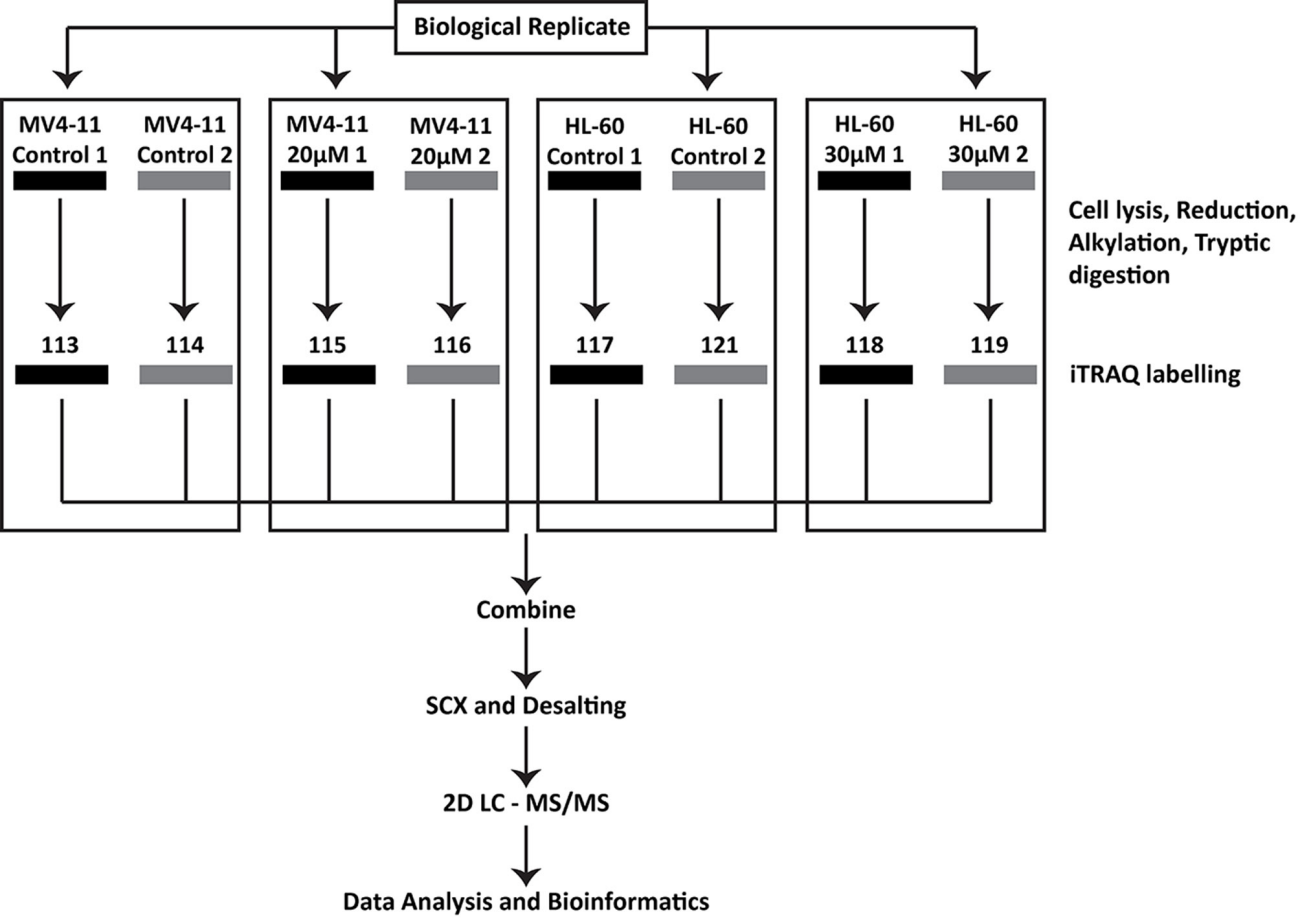
Samples generated for iTRAQ labelling and proteomic analysis Proteins were extracted from untreated control samples and 48 h genistein treated (20μM for MV4-11 and 30μM for HL-60) samples for both cell lines. Biological replicates were included for each cell lines, and all eight protein samples were reduced, alkylated and tryptic digested before labelling for 8-plex iTRAQ analysis as shown in the figure. The samples were then pooled and processed before subjecting to 2D LC-MS/MS analysis.

Proteomic profiling of the cells was performed by employing the 8-plex iTRAQ strategy. The samples were prepared as biological replicates shown in Figure 1. Significantly altered proteins were selected, and a total of 692 uniquely regulated proteins were identified from both AML cell lines. The breakdown on the distribution of the identified proteins showed that 436 and 410 proteins were significantly regulated in MV4-11 and HL-60 respectively (Figure 4). In addition, 267 proteins were found to have increased abundance in the two AML cell lines while 425 proteins showed decreased abundance. There was moderate overlap of proteins between the two cell lines for both up-regulated and down-regulated proteins. This could indicate similarity in the mechanism of action of genistein on both AML cell lines. However, there were still many proteins, which were not similarly altered in both cell lines, implying possible divergent effects by genistein treatment.

**Figure 2:**
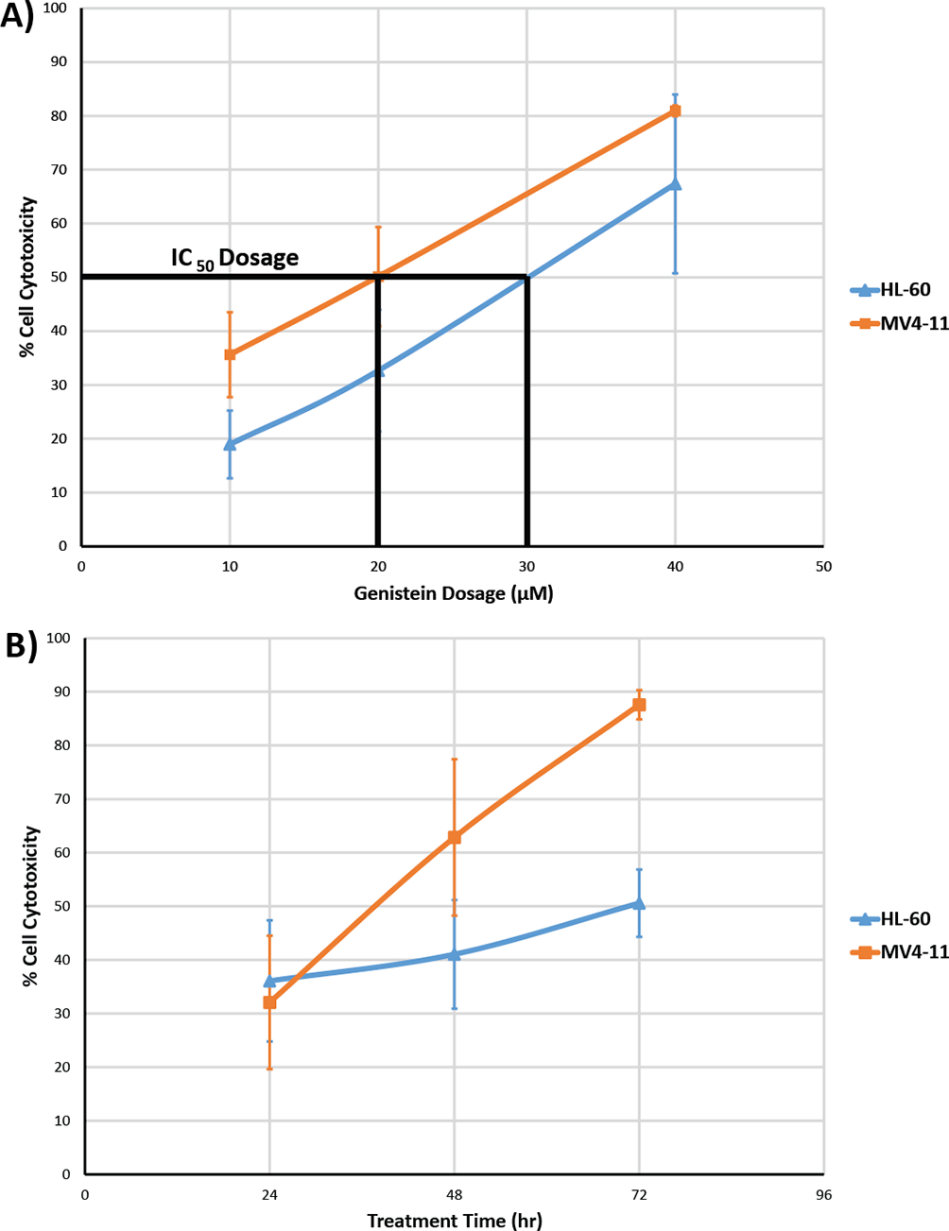
Cell cytotoxicity of genistein on MV4-11 and HL-60 cells Studies were conducted on both the (A) dose dependent cytotoxic effects (48 h) and (B) time dependent cytotoxic effects (20μM for MV4-11 and 30μM for HL-60) of genistein treatment on the two AML cell lines. The total number of cells at each dosage and time duration was counted using trypan blue exclusion staining to quantify the cytotoxic effect of genistein on the two AML cell lines. The IC_50_ for MV4-11 is approximately 20μM and HL-60 is approximately 30μM. The results show that genistein had an enhanced cytotoxic effect on MV4-11 as compared to HL-60 cells. The data shown is an average of a triplicate of experiments. Error bars represent standard deviation.

**Figure 3:**
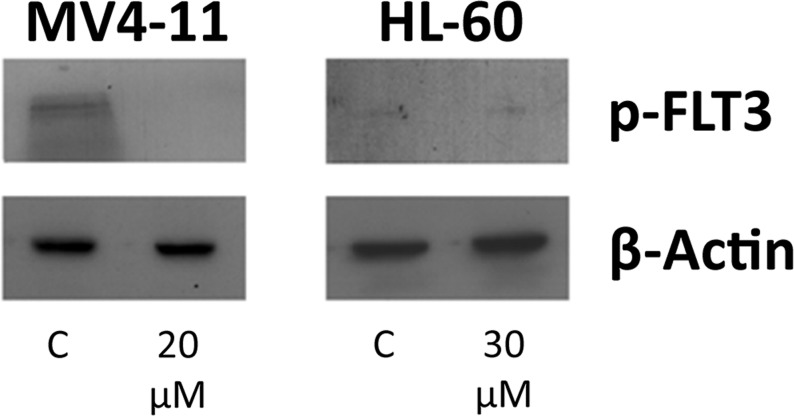
Genistein inhibits the activation of FLT3 protein Genistein blocks the constitutive phosphorylation of FLT3 in the MV4-11 cells, highlighting its function as an FLT3 inhibitor. HL-60 cells does not have the FLT3-ITD mutation, thus the activation of FLT3 is not observed in both the control (C) and the genistein-treated samples.

**Figure 4:**
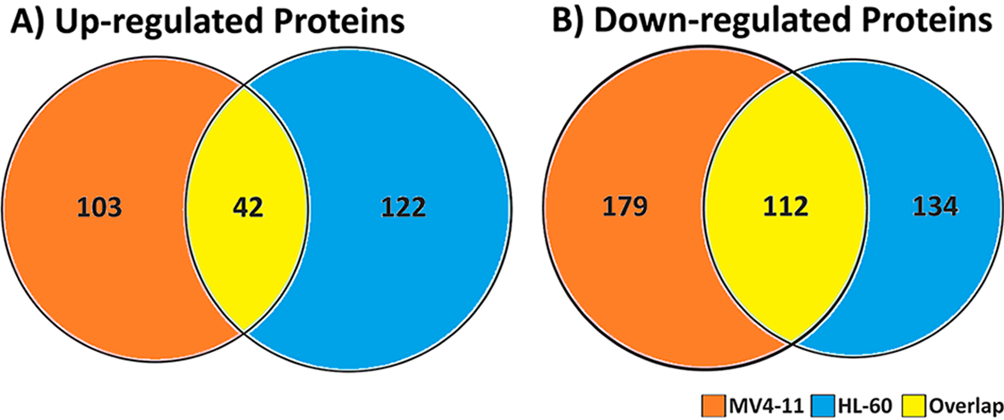
Comparison of the regulated proteins after genistein treatment of M4-11 and HL-60 cells identified from our iTRAQ-based proteomic study A total of 436 significantly regulated proteins were identified from MV4-11 cells and 410 proteins from HL-60 cells. A combined total of 692 uniquely regulated proteins were identified from both AML cell lines. A) A total of 267 proteins were identified to have increased abundance between the two AML cell lines. B) A total of 425 proteins were identified to have decreased abundance between the two AML cell lines. The data implies a moderate degree of overlap between the two cell lines, while also signifying unique regulation of a subset of proteins in each individual cell lines.

Since the samples were run as biological duplicates, it is critical to assess the reproducibility of the data. A high level of concordance between the duplicates would bolster the confidence in the data, and also lend credibility to the sample preparation methodology and the downstream mass spectrometric analysis. The scatter plot analysis of the duplicates was performed, and the R^2^ value of the trend line was used as a measure of reproducibility. As seen in Supplementary Figure 1, the duplicates exhibited high level of conformity, validating the soundness of the data.

### Genistein regulates crucial pathways in MV4-11 and HL-60 cells

**Figure 5:**
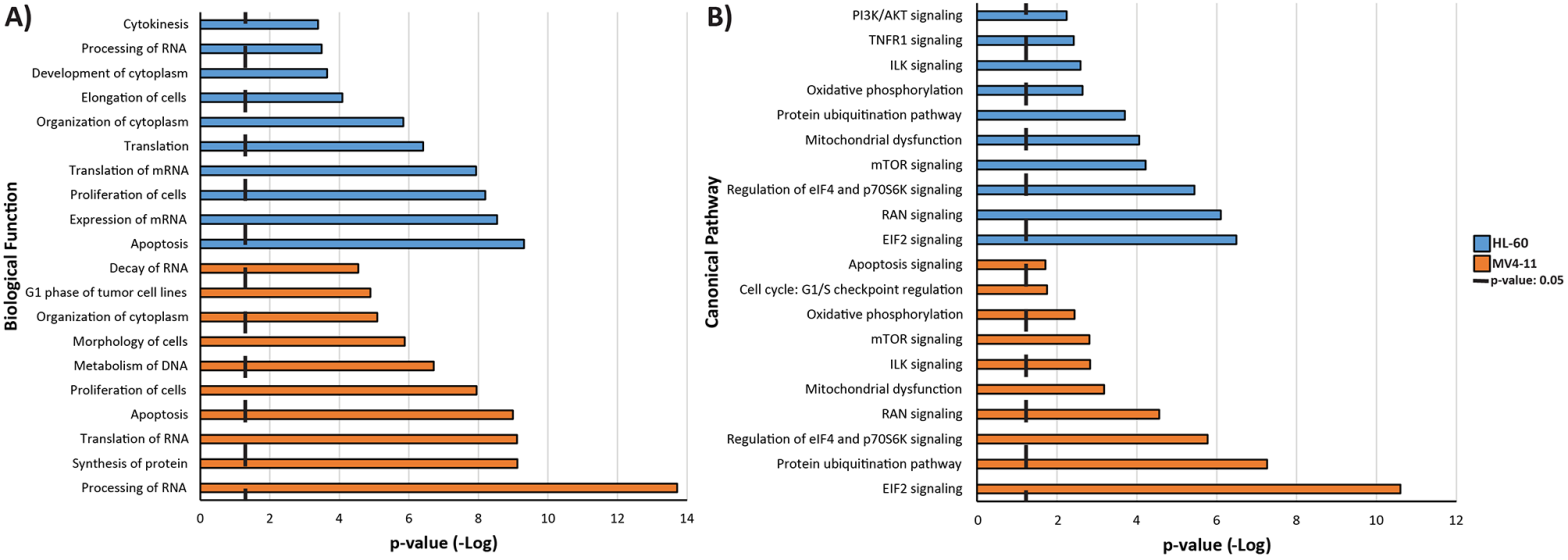
Bar chart showing the potential. A) biological function and B) canonical pathways involved in the proteome regulation by genistein treatment in MV4-11 and HL-60 cells The data were obtained after data analysis with the final list of significantly regulated proteins in IPA software. In accordance to the high degree of overlap of regulated proteins between the two cell lines, the biological functions observed were generally similar although minor differences show a potential divergence in genistein effect between the two AML cell lines.

Bioinformatics analysis was performed on the proteomics data using the IPA software. Potential biological functions regulated by genistein in MV4-11 and HL-60 were identified as shown in Figure 5A. A number of functions are common to both the cell lines. The general trend occurring in the two AML cell lines after genistein treatment is the effect on protein synthesis. Biological functions such as processing of RNA, synthesis of protein, translation of RNA, expression of mRNA, and translation of mRNA points to the possibility that protein synthesis is altered after genistein treatment on both AML cell lines. In addition, cell death of both AML cell lines seems to be triggered by apoptosis in both AML cell lines after genistein treatment. Furthermore, there are implications of cell cycle disruption occurring as observed from the presence of regulation in proliferation of cells, with MV4-11 cells showing biological processes regulating the G1 phase of tumor cell lines.

Figure 5B lists the potential canonical pathways, which were regulated in the two AML cell lines after genistein treatment. These potential pathways identified were reflective of the biological functions found modulated by genistein as shown in Figure 5A. There were significant similarities between the two cell lines, indicating a consistent mechanism of action employed by genistein. Majority of the canonical pathways identified is again related to protein synthesis in both AML cell lines such as EIF2 signaling [28], and mTOR signaling [29]. Regulation of eIF4 and p70S6K signaling is part of the mTOR signaling pathway. Canonical pathways such as RAN signaling [30], ILK signaling [31] and cell cycle: G1/S checkpoint regulation are involved in cell proliferation and cell cycle progression, similar to the biological functions identified in our study. Interestingly, ILK signaling is known to be involved in G1 phase cell cycle progression [32], which was also identified in our MV4-11 dataset. Furthermore, ILK signaling is also implicated in G2/M phase cell cycle arrest [33].

### Protein synthesis is negatively regulated by genistein via mTOR signaling

**Figure 6:**
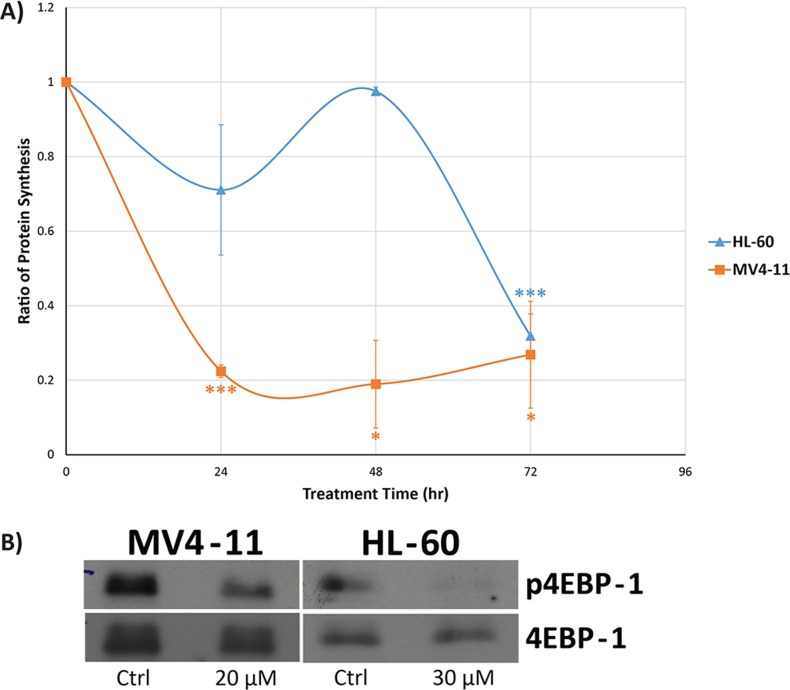
Genistein treatment results in decreased protein synthesis in MV4-11 and HL-60 cells A) Protein synthesis was analyzed using metabolic labelling, and there is an immediate and consistent drop in the protein synthesis rates in MV4-11 cells. However, a delayed effect is observed in the HL-60 cells, where significant decrease in protein synthesis was observed after 72 h of genistein treatment. The data shown is the mean of a triplicate of experiments. * indicates *p<0.05;* *** indicates *p <0.001.* Error bars represent standard deviation. B) Western blot analysis of mTOR pathway, which is the key pathway in regulating protein synthesis and is reported to be regulated from our iTRAQ analysis. Our result shows the reduced phosphorylation of the protein 4EBP-1, where the unphosphorylated form binds to eIF4E protein and inhibits its translational activity. This results in the decreased protein synthesis, in accordance to our protein synthesis assay.

From our quantitative proteomics analysis results shown in the previous section, protein synthesis seems to be greatly affected in both MV4-11 and HL-60 cells after genistein treatment. Thus, we measured the production of nascent protein using click chemistry approach. Our results show that genistein was able to reduce the level of protein synthesis in both MV4-11 and HL-60 by up to 80% of the control level (Figure 6A). Such an emphatic response at the translational level to genistein is a very interesting observation and previously unreported. However, it is important to note that the effect was manifested at different time points in both the cell lines. MV4-11 cells were quick to respond to genistein treatment and exhibited a drop in protein synthesis levels as early as 24 h after drug treatment, sustaining the reduction in protein levels up to 72 h. However, the HL-60 cells showed prolific reduction only at 72 h, with little or no response at the earlier time points.

From our IPA results, we identified canonical pathways involved in mTOR signaling as mentioned in the previous section. The protein, 4EBP-1, is the downstream effector of mTOR signaling. It binds to the eukaryotic translation initiation factor 4E (eIF4E) protein to prevent eIF4E from triggering protein synthesis. The activation of mTOR pathway can lead to the phosphorylation of 4EBP-1, releasing it from eIF4E. The free eIF4E will then initiate protein synthesis. Thus, if protein synthesis was perturbed by genistein via targeting mTOR pathway, the phosphorylation level of 4EBP-1 should show a reduction in levels. Indeed, our western blot analysis indicated a reduced phosphorylation of 4EBP-1 after genistein treatment in both MV4-11 and HL-60 cells (Figure 6B). This highlights that mTOR pathway could be down-regulated by genistein treatment, an observation not previously reported.

### Genistein induces apoptosis in both MV4-11 and HL-60 cells via caspase activation

**Figure 7:**
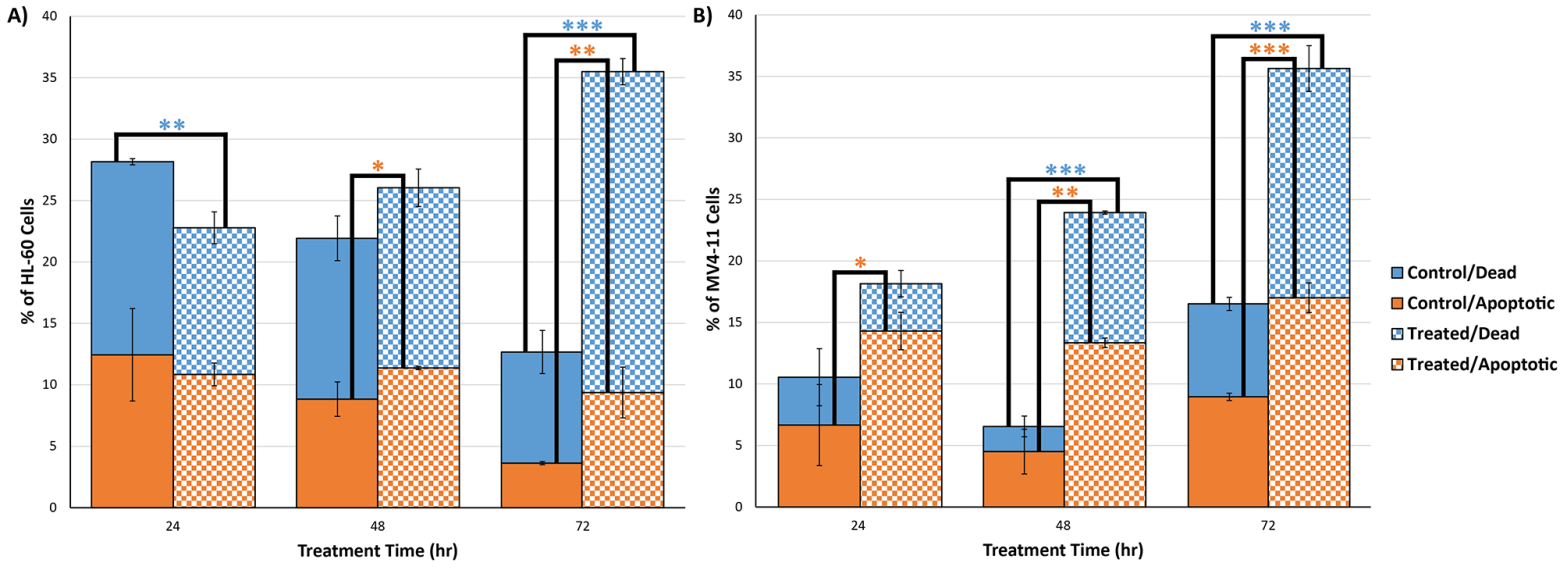
Genistein causes apoptosis in both (A) HL-60 and (B) MV4-11 cells Genistein treatment causes an increase in the proportion of the apoptotic and dead cells in both the cell lines. As is the case with the other observations, the apoptotic response in the MV4-11 cells is faster. The data shown is the mean of triplicate experiments. * indicates *p<0.05;* ** indicates *p<0.01;* *** indicates *p<0.001.* Error bars represent standard deviation.

The cells were assayed for apoptosis using flow cytometry based Annexin-V FITC staining. Genistein was found to induce apoptosis in both the cell lines. As Figure 7 indicates, an accumulation of apoptotic cells was observed in both the cell lines. It was also observed that the drug caused an increase in the dead cell population. These cells stained positively for both PI and FITC. However in this subpopulation of cells, it is not possible to discern those that died by apoptosis from ones that died by necrosis. More apoptotic/dead cells were observed after 72 h of genistein treatment than other time points, which is concordant with our *in vitro* cytotoxicity assays, which indicated a peak effect of genistein at 72 h in both the cell lines (Figure 2B). Thus, it is apparent that genistein induces apoptosis in both the cell lines.

**Figure 8:**
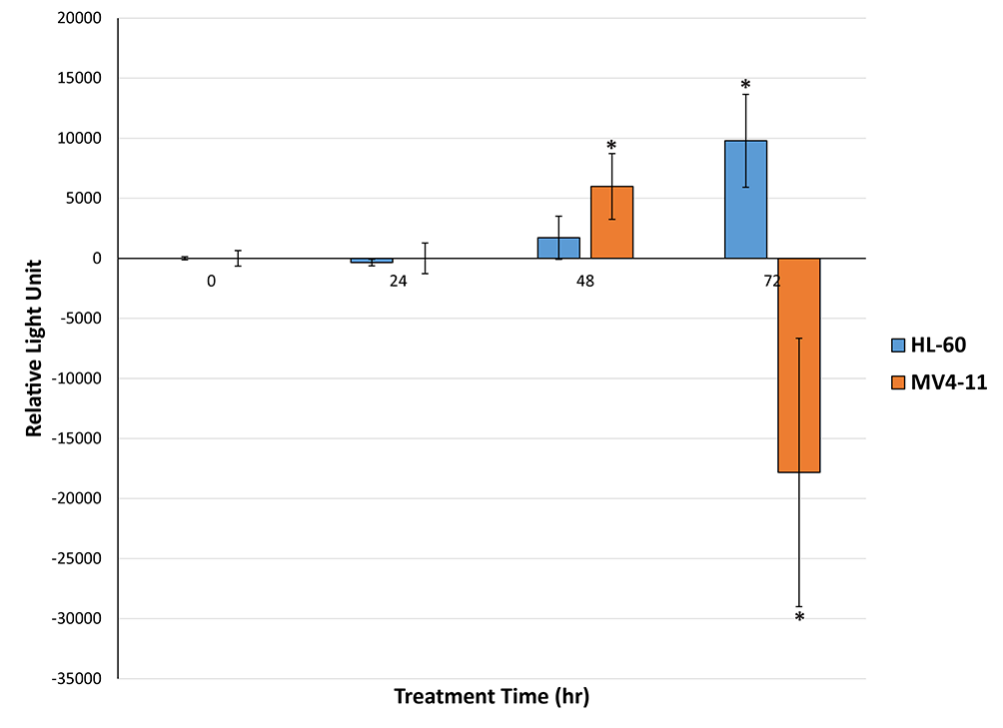
Genistein exerts caspase mediated apoptosis on both HL-60 and MV4-11 cells Genistein adopts a caspase mediated approach to apoptosis in the HL-60 cells as implied by the significant increase in caspase levels after 72 h of treatment. However the apoptotic effect of genistein is much stronger in MV4-11 cells as the significant increase in caspase levels was observed after 48 h of treatment. The drastic drop in caspase levels for MV4-11 cells after 72 h treatment could indicate late stage apoptosis. * indicates *p<0.05*. Error bars represent standard deviation.

In order to understand the mechanism of apoptosis caused by genistein treatment in AML, the caspase 3/7 levels of AML cells treated with genistein were assayed. Caspase activation is a major mode of apoptosis induction in cells. Our results show that caspase activation was present in both MV4-11 and HL-60 cells (Figure 8). However, significant caspase activity was observed after 48 h of genistein treatment in MV4-11 cells while significant caspase activity was only observed after 72 h of genistein treatment in HL-60 cells. This result is consistent with our Annexin-V-FITC results, which showed a significantly higher population of apoptotic cells in MV4-11 cells than in HL-60 cells after 48 h of genistein treatment. Furthermore, there was a drastic drop in caspase activation in MV4-11 cells after 72 h of treatment. This could be a result of a majority of cells already entering late stage apoptosis or necrosis leading to a decline in the levels of caspase. This is again consistent with our Annexin-V-FITC results which show a general higher number of apoptotic cells in MV4-11 cells than in HL-60 cells. This highlights a stronger apoptotic effect of genistein on MV4-11 cells than on HL-60 as reflected by the lower IC_50_ dosage of genistein.

### Different modes of cell cycle arrest caused by genistein between MV4-11 and HL-60 cells

In our IPA results, we identified our regulated proteins to be involved in cell proliferation in both MV4-11 and HL-60 cells. In addition, regulated proteins were also found to be involved in G1 phase of tumor cell lines in MV4-11 cells and cytokinesis in HL-60 cells. Furthermore, both RAN and ILK signaling which we identified from our canonical pathway search are also implicated in cell cycle progression as mentioned earlier. This highlights the potential implication of cell cycle modulation by genistein treatment.

**Figure 9:**
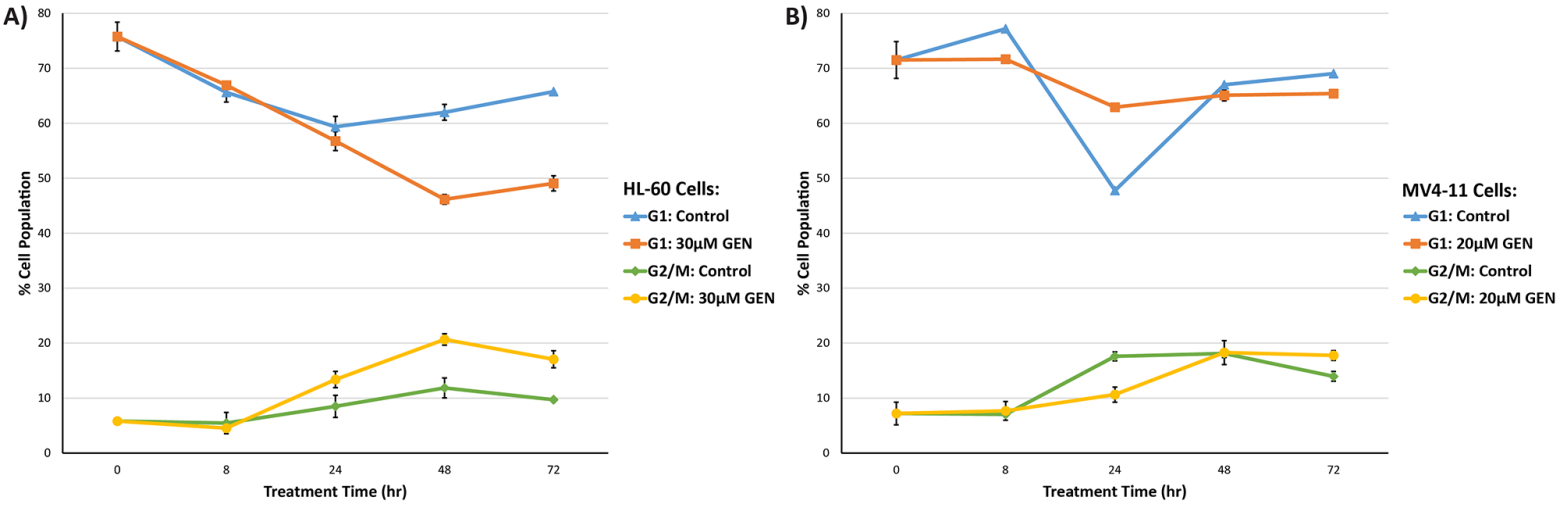
Cell cycle regulation by genistein in (A) HL-60 (B) MV4-11 cells Propidium iodide based flow cytometric analysis of cell cycle indicated divergent effects of genistein between the two AML cell lines. In HL-60 cells, cell cycle arrest occurs at G2/M phase after 48 h of genistein treatment. However, in MV4-11 cells, cell cycle arrest occurs at the G1/S phase after 24 h of genistein treatment. Error bars represent standard deviation.

Our cell cycle analysis depicted in Figure 9 clearly elucidates that genistein induces G2/M arrest in HL-60 cells. The drug causes a relative accumulation of cycling cells in the G2/M phase of the cell cycle with time, thereby blocking cell division and cellular proliferation. However, in the case of the MV4-11 cells, a low degree of sensitivity to genistein treatment was observed in the cell cycle analysis. The MV4-11 cells showed an accumulation of cells in the G1/S phase after 24 h treatment of genistein, and a G2/M arrest after 72 h, in stark contrast to the observation in the genistein treated HL-60 cells. This reveals a fundamental difference in the cell cycle regulation by genistein between the two cell lines.

## DISCUSSION

The anti-proliferative effect of genistein, an isoflavone, on tumor cells has been widely reported and touted as an alternate therapy for treating patients with AML [22]. Various studies have attempted to elucidate the mechanism of action of genistein in AML such as the one by Shen *et al.* [21]. Yet, the mechanism of action of genistein is still vague and subjected to speculation. Also, none of the earlier studies have attempted a comprehensive study on the effects of genistein encompassing cell lines with different genetic mutation status. Hence, our study is the first to evaluate the effects of genistein treatment on AML cells, using a two cell line model, with the cell line MV4-11 containing FLT3-ITD mutation and HL-60 with the wildtype FLT3 gene. The FLT3-ITD mutation was chosen due to its prominence among patients suffering from AML (~23% of all AML incidences) as well as the lack of effective drugs for these patients [10, 12].

The *in vitro* cytotoxicity assays demonstrated the potent inhibitory effect of genistein on both AML cell lines. The MV4-11 cells were seen to be slightly more responsive to genistein treatment than HL-60, as typified by the lower IC50 dosage values. The tyrosine kinase inhibitory activity of genistein has been well established decades ago, and genistein is also recently found to be a FLT3 inhibitor [27]. Our study also validated that genistein could inhibit the constitutive phosphorylation of FLT3 in the MV4-11 cell line (not previously used with genistein treatment; Figure 3). FLT3 inhibitors usually have specific cytotoxicity towards cells harboring the FLT3 mutation. Drugs such as ABT-869—a FLT3 inhibitor—have little or no effect on cells containing wild-type FLT3 gene [34, 35]. In this context, the anti-oncogenic property of genistein has a significant advantage. This widens the scope and application of genistein, and paves the way for its use as a generic AML therapy. Hence, it would be beneficial to improve the knowledge of the mechanism of action of genistein.

Subsequently, we profiled the proteome of MV4-11 and HL-60 cells treated with their respective genistein IC dosage, comparing them with their respective untreated cells. The iTRAQ platform used for the study was designed in a manner to accommodate the labelling of 8 samples in parallel. This allowed us to label each of the samples in biological duplicates, thus enhancing the statistical power during analysis, leading to a higher level of veracity in our data. The IPA analysis helped uncover a lot of pathways deemed significant in explaining genistein's anti-leukemic effects. The canonical pathways identified as regulated in both MV4-11 and HL-60 showed significant similarity and correlated well with the identified biological functions.

### Down-regulation of mTOR and reduced protein synthesis

Delving deeper into the data, we observed that alteration in the process of protein synthesis is consistently predominate between MV4-11 and HL-60 cells. This might be indicative of an arrest on protein translation, synthesis and processing by genistein treatment, leading to the inhibition of cellular growth and proliferation. In accordance with this line of thought, mTOR signaling and protein ubiquitination pathways were also found to be regulated by genistein in both AML cell lines (Figure 5B).

mTOR has been an attractive target for cancer in general, with hematologic malignancies in particular [36–38]. This pathway controls important biological functions that include protein translation and cellular growth, which are of special significance in explaining the anti-leukemic property of genistein [39, 40]. Western blot analysis of p-4EBP-1, the downstream effector of mTOR validated the inhibitory effect of genistein on the mTOR signaling axis (Figure 6B). The phosphorylation of 4EBP-1 is essential for the control of translation initiation. Earlier studies have shown that genistein exhibited inhibitory effects on protein synthesis in leukemia cells, mediated by eIF2α kinase [41].

Thus, there is growing evidence to suggest that genistein causes a decrease in the synthesis of proteins mediated by the arrest of the mTOR pathway. Our nascent protein synthesis assay using Click™ chemistry-based approach elucidated that genistein treatment drastically reduced the overall protein synthesis rate in MV4-11 cells (Figure 6A). Contrastingly, in the case of HL-60, the effect was most pronounced only at 72 h, with the other time points displaying milder reduction in protein synthesis rates. Thus, our study highlights mTOR arrest and subsequent protein synthesis inhibition as key events orchestrated by genistein treatment.

### Apoptotic cell death by genistein

Annexin-V-FITC assay—a technique which has the capability to specifically differentiate between apoptotic and necrotic cells—showed that genistein induces apoptosis in both MV4-11 and HL-60 cells, with the effect peaking at 72 h (Figure 7). This observation is further backed up by the trypan blue counting results, which demonstrate the cytotoxic effect of genistein treatment in both AML cell lines. Once we established that apoptosis is the main mode of cell death employed by genistein, we probed deeper into the mechanism of apoptosis initiated by the drug. Caspase activation is a hallmark of apoptosis.

The caspase 3/7 levels were assayed in cells treated with genistein. We found that both MV4-11 and HL-60 underwent apoptosis via caspase activation (Figure 8). However, significant caspase activity was observed earlier in MV4-11 cells after 48 h genistein treatment, compared to significant activation observed in HL-60 cells after 72 h genistein treatment. Thus, MV4-11 cells were observed to be more sensitive to genistein treatment than HL-60 as highlighted by the earlier induction of caspase activity. The drastic decrease in caspase activity in MV4-11 cells after 72 h genistein treatment could be due to the rapid degradation of the caspase [42] after its initial activation. This further suggests the early induction of caspase activity in MV4-11 cells.

### Differential effects on the cell cycle

Genistein has been known to arrest the cell cycle progression of AML cells, thus inhibiting the growth and proliferation of cells [21, 43]. Literature suggests that genistein could induce a G2/M arrest in AML cells, and HL-60 in particular. In our study, we did find that genistein caused a G2/M arrest in HL-60 cells. However, in the case of MV4-11, this phenomenon was absent. There have been previous instances of drugs exerting contradicting effects on the cell cycle based on the presence and absence of FLT3-ITD mutations.

In a study conducted on series of AML cells with and without this mutation, it was found that the drug PKC412—a FLT3 inhibitor—arrested the cells with FLT3 mutation at the G1 phase, while those without the FLT3 mutation at the G2/M phase of the cell cycle [12, 13]. PKC412 was highly cytotoxic to cells containing the mutation, but not very effective against the ones with wild-type FLT3 gene. This observation is similar to our results, where genistein seems to exert more potent cytotoxic effect on MV4-11 than HL-60 cells. Therefore, our study highlighted that the FLT3 mutational status in AML cell lines could determine the regulatory effect of genistein on cell cycle progression.

Using PathVisio [44] and WikiPathways database [45], we analyzed the possible differences in the cell cycle pathway between MV4-11 and HL-60 cells using our final protein list. From the pathway analysis we have obtained, we identified proteins like retinoblastoma protein (RB1), histone deacetylase 1 and 2 (HDAC1 and HDAC2), and DNA-dependent protein kinase catalytic subunit (PRKDC) being regulated differently between the two cell lines (Supplementary Figure 2). From literature, it is reported that RB1 is able to induce cell cycle arrest at G1 phase in lymphoblastic cells after treatment with glucocorticoids [46]. This difference caused by genistein treatment on the two cell line might explain for the differences seen in the cell cycle regulation by genistein in both the cell lines, but further work is required to justify this observation, which is beyond our current research scope.

### Examining the role of FLT3

In spite of the apparent similarities in the mechanism of action of genistein in the two model cell lines, the inherent differences surfaced when we probed deep. Processes such as protein synthesis arrest and caspase-dependent apoptosis are common to both cell lines. The causal factors such as mTOR arrest is also shared by both. However the degree of regulation and time of onset is different for the two models. Previous literature indicates that genistein induces cell cycle arrest and apoptosis in AML. Our study corroborates this fact. However, it is very important to note that we found a major difference in the mode of cell cycle regulation between the two AML cell lines.

Although baffling, such a difference in the effects of genistein could be explained by incorporating its inhibitory effect on the phosphorylation status of FLT3 in MV4-11. This intrinsic property of genistein provides it with an additional method to exert cytotoxic effect on AML cells. Since FLT3 is a very important kinase for cell growth and proliferation, it has a number of downstream effectors. Thus, a modification to its constitutively activated status would elicit several downstream responses. Such responses would obviously be unique to MV4-11 which contains the mutated FLT3 gene and would not be observed in HL-60 which has the wild-type gene.

It is also possible that the inhibitory effect of genistein on the FLT3 phosphorylation in MV4-11 makes the cells highly responsive to the cytotoxic effects of the drug and hastens the biological processes such as protein synthesis arrest and apoptosis. Hence, the fact that genistein acts as a FLT3 inhibitor in AML provides a plausible explanation to the divergent effects exerted by genistein on certain biological processes in these cell lines. Therefore, the differences in protein changes between the two cell lines that we have identified might allow us to further explore how these divergent effects result, providing us a platform to further explore and elicit genistein's mechanism of action.

## CONCLUSION

**Figure 10:**
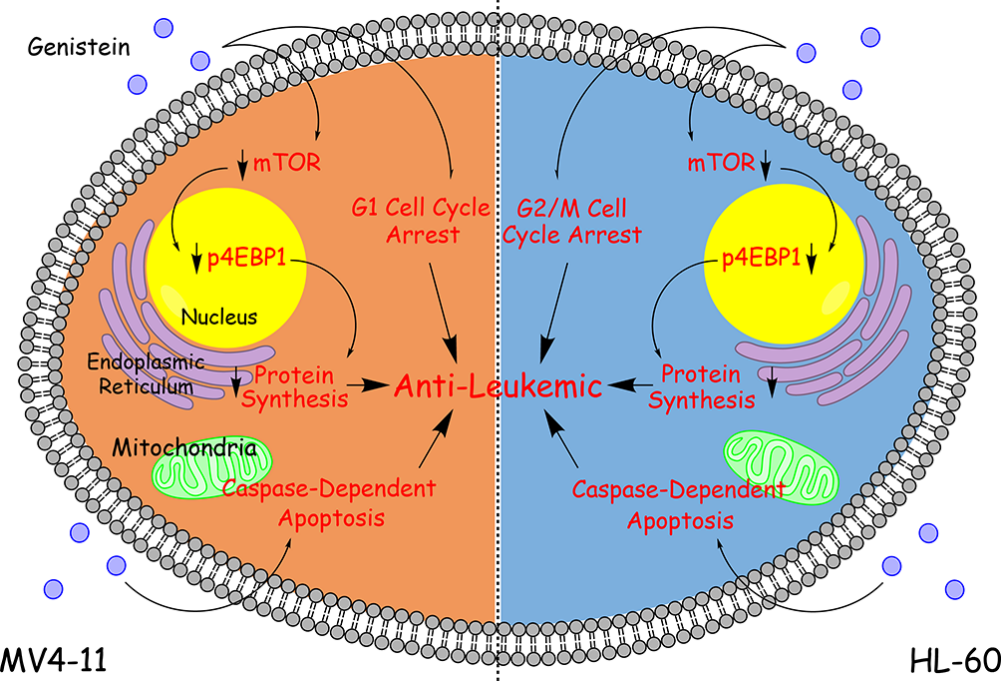
Summary of the effects of genistein on AML Modular representation of the mechanism of action of genistein in AML, based on empirical evidences. Genistein exerts its anti-leukemic effect in AML cells via down-regulation of the mTOR pathway leading to repression of protein synthesis, induction of cell cycle arrest, and elevation of apoptosis. The only difference in the mode of action by genistein between the two AML cell lines is that G1 cell cycle arrest is triggered in MV4-11 cells but G2/M cell cycle arrest is triggered in HL-60 cells.

In conclusion, we have characterized the effects of genistein on AML in a comprehensive manner using a two-cell line model. Genistein has been demonstrated to have anti-leukemic effect on two AML cells with different genetic makeup. We have also confirmed that genistein is a FLT3 inhibitor. We have shown that mTOR pathway was arrested by genistein treatment, leading to protein synthesis inhibition. Caspase-dependent apoptosis was also triggered in both AML cell lines after genistein treatment, with caspase activity peaking earlier in MV4-11 cells than HL-60. In addition, we identified that genistein employs a divergent mechanism to initiate cell cycle arrest between MV4-11 and HL-60 cells. On the weight of our results, we espouse an empirical model to encapsulate the mode of action of genistein in AML (Figure 10). With many more protein changes that have been identified by our studies and interesting insights we have discovered as seen in Supplementary Figure 2, we hope to further construct a clearer mechanism of action which genistein works on in future works. A better understanding in this area of genistein would further demonstrate the attractiveness of genistein in AML therapy and even in other types of cancers.

## MATERIALS AND METHODS

### Cell Culture

The cell lines used in this study were MV4-11 and HL-60. The MV4-11 cells carry a FLT3-ITD mutation, while the HL-60 cells contain the wild-type FLT3 gene. Both the cell lines were cultured in complete RPMI media supplemented with 10% FCS in a humidified incubator at 37°C with 5% CO_2_. Cells were grown in T-75 tissue culture flasks with aseptic conditions strictly maintained.

### *In vitro* cytotoxicity assay

Genistein was purchased from Sigma-Aldrich (St. Louis; MO, USA), and stock solutions of 100mM were prepared. The aliquots were stored at −20°C. The final concentration of DMSO in the *in vitro* assays was maintained at <0.05%. The cytotoxicity of the drug was assessed using trypan blue exclusion dye (Sigma-Aldrich). Cells were treated with genistein at various dosages of 10μM, 20μM, 40μM, and for different time points of 0 h, 24 h, 48 h and 72 h. The total number of viable cells in the treated and control cells were calculated to assess the cytotoxicity of genistein.

### Protein extraction and sample preparation for iTRAQ™ labelling

The proteome profiles of cells treated with genistein were generated using the 8-plex iTRAQ labelling technique (AB SCIEX; Foster City, CA, USA). The cells were harvested and lysed for protein extraction with 0.5M Triethylammonium bicarbonate (TEAB), pH 8.5 containing 1% SDS and protease inhibitor (Complete Protease Inhibitor Cocktail Tablets, Roche; Basil, Switzerland). The cells were incubated with this lysis buffer at room temperature for 15 min and subsequently at 100°C for 20 min. Protein separation was achieved by centrifuging at 4°C for 1 h at maximum speed of 14,000 rpm. A hundred μg of each sample was used for iTRAQ labelling.

The protein samples were diluted ten times with 0.5M TEAB, pH 8.5 and subjected to reduction with 5mM tris-(2-carboxyethyl) phosphine at 60°C for 1 h followed by alkylation with 10mM methyl methanethiosulfonate (MMTS) for 10 min, and then diluted to 0.05% (w/v) SDS. The samples were then digested with trypsin (AB SCIEX) at 37°C for 16 h. Following this, each tryptic digest was labelled for 2 h with one of the eight isobaric amine-reactive tags at room temperature. The samples were prepared as biological duplicates and processed using the labelling pattern depicted in Figure 1.

### Cation exchange purification and desalting of labelled samples

These iTRAQ-derivatised samples were then pooled and passed through a strong cation exchange (SCX) cartridge as recommended by the manufacturer (AB SCIEX). The bound peptides were eluted with 10mM KH_2_ PO_4_ in 25% ACN/350mM KCl, pH 3. This eluate was desalted using a Sep-Pak® C_18_ cartridge (WATERS; Milford, MA, USA) before subjecting to vacuum-drying and reconstitution with 50μL of 5mM KH_2_ PO_4_ buffer containing 5% ACN, pH 3 for 2D-LC separation.

### 2D LC separation of iTRAQ™-labelled peptides and mass spectrometry analysis

The 2D LC separation of our iTRAQ-labelled samples was carried out as reported previously [23]. Ninety-six μg (via multiple injections) of the labelled peptide mixture was injected using the micro-pickup mode into a Zorbax Bio-SCX II column (Agilent; SantaClara, CA, USA). A total of 78 fractions were eluted (2 min each) and collected on a 96 well v-bottom plate. The eluted fractions were subsequently combined to 11 fractions and then desalted with Sep-Pak^®^ tC18 μElution Plate (WATERS) using a vacuum manifold (Millipore; Billerica, MA, USA) in accordance to the manufacturer's recommendations before second-dimension reversed-phase (RP) chromatography. The MS analysis was performed using a 5600 TripleTOF analyzer (QqTOF; AB SCIEX) in Information Dependent Mode.

### Database search and protein identification

Protein identification and relative iTRAQ quantification were performed with ProteinPilot™ Software 4.5 (AB SCIEX) using the Paragon™ algorithm for the peptide identification which was further processed by Pro Group™ algorithm, where isoform-specific quantification was adopted to trace the differences between expressions of various isoforms. The Pro Group™ Algorithm calculates protein ratios using only ratios from the spectra that are distinct to each protein or protein form and thus eliminates any masking of changes in expression due to peptides that are shared among proteins.

User defined search parameters were as follows: (i) Sample Type: iTRAQ™ 8-plex (Peptide Labelled); (ii) Cysteine Alkylation: MMTS; (iii) Digestion: Trypsin; (iv) Instrument: TripleTOF 5600; (v) Special Factors: None; (vi) Species: Homo sapiens; (vii) ID Focus: Biological modifications; (viii) Database: Oct2012_uniprot_sprot. fasta (40468 proteins searched); (ix) Search Effort: Thorough; (x) FDR Analysis: Yes; (xi) User Modified Parameter Files: No. For iTRAQ™ quantitation, the resulting dataset was auto bias-corrected to account for variations due to unequal sample loading. A reverse database search strategy was adopted to estimate the false discovery rate (FDR) for protein identification. Global FDR = 1% was chosen as the cut-off threshold to generate the list of identified proteins.

### Selection of significantly altered proteins and Bioinformatics analysis

After the ProteinPilot search results were obtained, proteins with only a single unique peptide were filtered out before a protein ratio cut-off threshold of ≥ 1.3 and ≤ 0.77 was applied as determined previously [24]. Next, a two-sample *t*-test was performed to check whether the fold-change (Log_2_ of each protein ratio) of each protein derived from the biological replicates was significantly (*p* ≤ 0.05) different from 0. Lastly, since each cell line duplicates yields four ratios, proteins with 3 or more ratios constantly above or below our cut-off thresholds of ≥ 1.3 and ≤ 0.77 were added to our final protein list for MV4-11 and HL-60 cells (Supplementary Table 1 and 2 respectively). However, proteins with three ratios above ≥ 1.3 and one ratio ≤ 0.77, and vice versa were not included in our final list due to possible discrepancy. The unfiltered protein list as well as the protein list at each step of filtering can be found in Supplementary Table 3 and 4 respectively.

Subsequently, our final protein lists were uploaded onto the Ingenuity Pathway Analysis (IPA) software (Ingenuity Systems; Redwood City, CA, USA). The software analyzed the datasets and identified the canonical pathways and biological functions regulated by genistein in the cell lines being investigated.

### Western Blot Analysis

Antibodies against p-FLT3, p-4EBP-1 and 4EBP-1 were obtained from Cell Signaling Technologies (Danvers, MA, USA). The western blotting protocol suggested by the vendors was followed.

### Quantification of nascent protein synthesis using Click chemistry

The amount of nascent proteins produced by the cells treated with genistein in comparison to the untreated cells was studied using the Click-iT^®^ Metabolic Labelling Reagents for Proteins and Click-iT Cell Reaction Buffer Kit (Molecular Probes, Invitrogen). Cells were treated with genistein and harvested at 0 h, 24 h, 48 h and 72 h time points.

The metabolic labelling reagent used was Click-iT AHA (L-azidohomoalanine), a methionine alternative. The cells were harvested and washed with 1x PBS. The cells were depleted of their methionine reserves by incubating them in methionine-free RPMI for 45 min. The cells were then transferred to the normal growth medium. fifty μM of AHA was added to the growth medium and the cells were incubated for 2 h. The cells were then harvested and fixed in 4% paraformaldehyde for 15 min. This was followed by permeabilization with 0.1% saponin for 15 min. 3% BSA was used to wash the cells.

The Click-iT reaction cocktail was prepared as per the manufacturer's guidelines. 0.5ml of the click-iT reaction cocktail was added to each sample and incubated for 30 min at room temperature. The cells were then washed once with 3% BSA, and the fluorescence was measured using a BD FACS Calibur flow cytometer, set to capture the fluorescence of Alexa Fluor 488 dye.

### Annexin V-FITC apoptosis detection

The cells were treated with genistein for the following time points: 0 h, 8 h, 24 h, 48 h, and 72 h. The cells were stained with annexin V-FITC and PI to detect apoptosis using the BD Pharmingen™ kit. The manufacturer's instructions were followed to label the cells. Single-stained controls were included to perform compensation. The BD FACS Calibur machine was used to perform the flow cytometry. Fifty-thousand relevant cells were collected in each case. The data was analyzed using the FlowJo software Version 7.6 (Tree Star Inc.; Ashland, OR, USA) to assess the percentage of cells undergoing apoptosis.

### Caspase 3/7 Assay

The drug treated MV4-11 and HL-60 cells were incubated for 0 h, 24 h, 48 h and 72 h and assayed for caspase mediated apoptosis. The treatments were carried out in 96-well plates. The cells were incubated with equal volumes of Caspase-Glo^®^ 3/7 reagent (Promega; Madison, USA) for 1 h along with the necessary controls, and the luminescence was measured.

### Cell Cycle Analysis

Cells were treated with genistein and harvested at 0 h, 8 h, 24 h, 48 h and 72 h time points after drug treatment. The cells were washed twice with 1× PBS and fixed in ice-cold 70% ethanol. The fixed cells were then stored in −20°C until further staining and analysis. The cells were stained with staining solution containing 0.1% Triton X-100 in PBS, 200μg/ml of RNaseA and 20μg/ml of propidium iodide (PI) for 30 min. The DNA content of the samples was studied by analyzing the samples on the CyAn Flow Cytometer (Dako, Denmark). Fifty-thousand relevant cells were collected in each sample. The DNA content distribution was obtained by analysis using the Summit 4.3 software (Dako).

## SUPPLEMENTARY MATERIAL FIGURES AND TABLES




