# Supplementary material for: Genistein exerts anti-leukemic effects on genetically different acute myeloid leukemia cell lines by inhibiting protein synthesis and cell proliferation while inducing apoptosis – molecular insights from an iTRAQ™ quantitative proteomics study
Source: Oncoscience. 2015 Feb 6;2(2):111–24. doi: 10.18632/oncoscience.120 (PMC4381704; doi:10.18632/oncoscience.120)
Supplement: Supplementary file 1 [file oncoscience-02-0111-s001.pdf]

# Genistein exerts anti-leukemic effects on genetically different acute myeloid leukemia cell lines by inhibiting protein synthesis and cell proliferation while inducing apoptosis – molecular insights from an iTRAQ™ quantitative proteomics study

## Supplementary Material

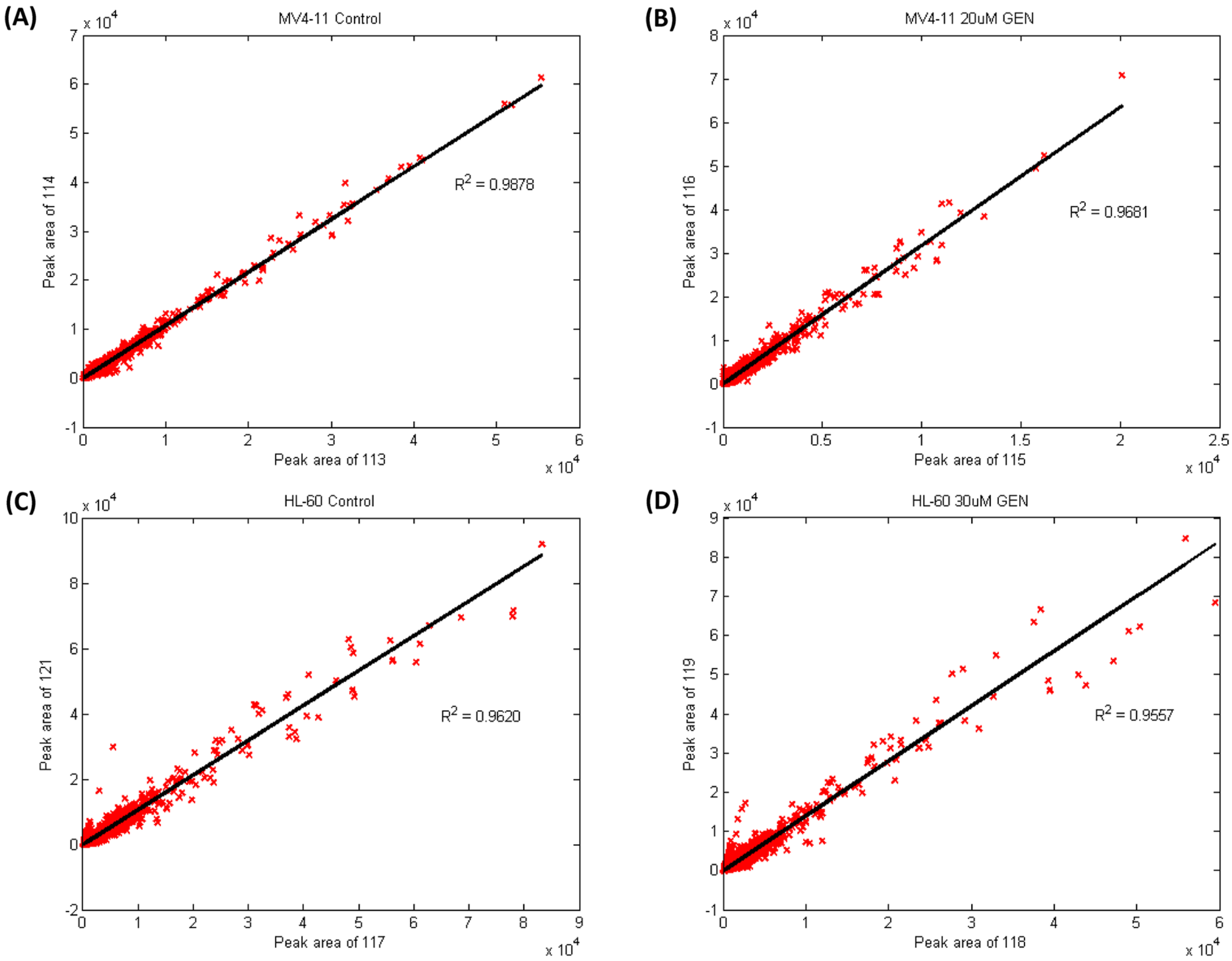


**Supplementary Figure 1: Regressional analysis of the scatter plots generated from the respective peptide peak areas from the various samples.**
The peak areas of each peptides, obtained from the generated petide summary using ProteinPilot (AB SCIEX) software, for each labelled samples were ploted in a scatter plot before conducting a regressional analysis to determine the $R^2$ value for each set of treatment duplicates: (A)MV4-11 Control; (B)MV4-11 cells treated with 20uM of genistein (GEN); (C)HL-60 Control; (D) HL-60 cells treated with 20uM of GEN. $R^2$ values closed to 1 in a regressional analysis usually shows close correlation between the two sets of data points compared. The generally high $R^2$ values across all sets of treatment duplicates demonstrate the high conformity for each samples.

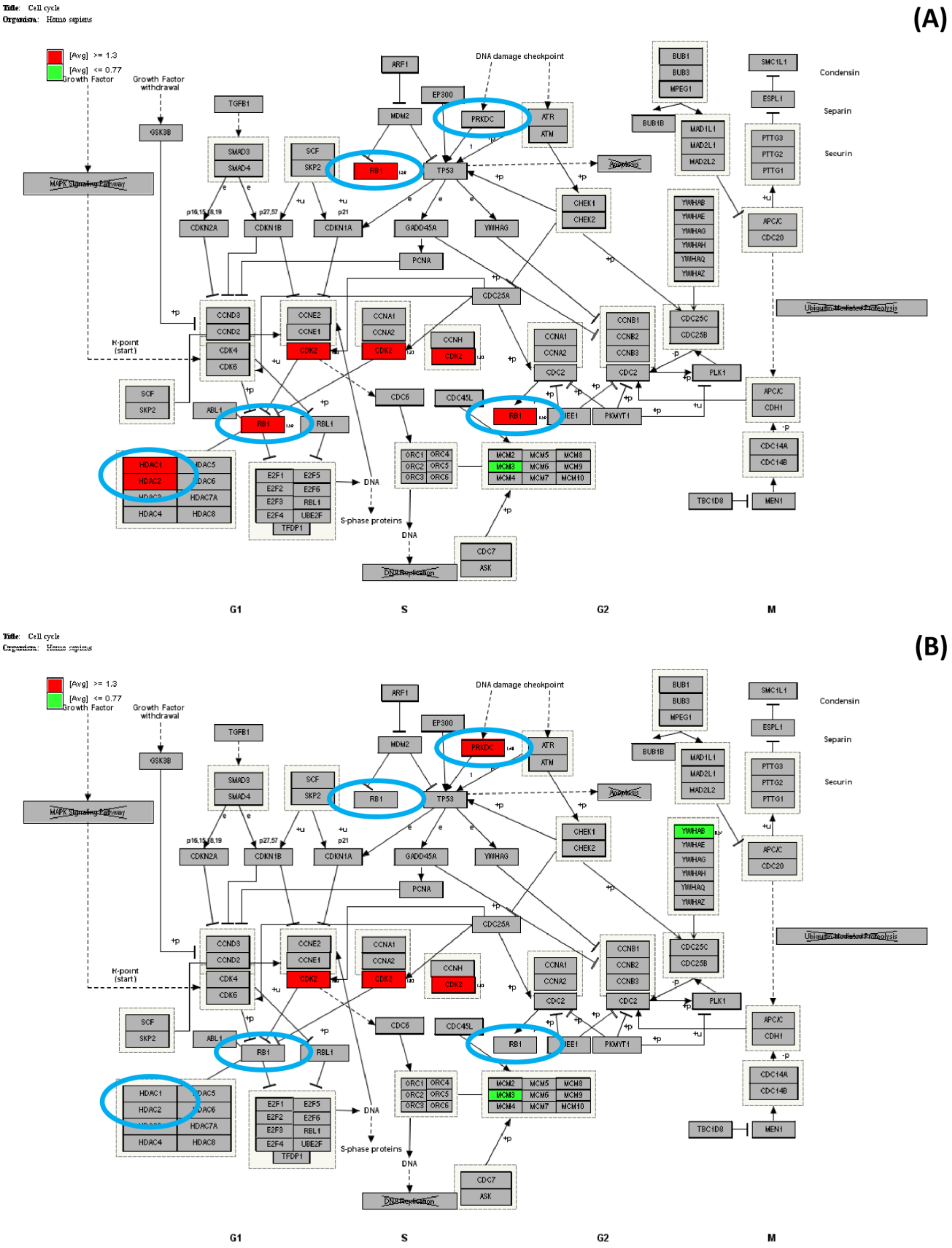


Supplementary Figure 2. Cell cycle pathway mapped with the iTRAQ$^{TM}$ data of MV4-11 (A) and HL-60 (B) cell lines. Blue circle highlights the difference in protein expression profile between MV4-11 and HL-60 cell lines after treatment with genistein. Retinoblastoma protein (RB1), Histone deacetylase 1 and 2 (HDAC1 and HDAC2) were up-regulated in MV4-11 cells but not in HL-60 cells. DNA-dependent protein kinase catalytic subunit (PRKDC) was up-regulate in HL-60 cells but not in MV4-11 cells.
